# The role of sulfonylurea plus basal insulin on glycemic variability compared to basal bolus regime in T2D patients

**DOI:** 10.1186/1758-5996-7-S1-A66

**Published:** 2015-11-11

**Authors:** Paula Vieira Freire, Erika Bezerra Parente, Cristal Peters Cabral, Andre Carvalho Yamaya, Caroline Schnoll, Vivian Roberta Ferreia Simões, Alessandra Muto, Manoel Carlos Sampaio de Almeida Ribeiro, João Eduardo Nunes Salles

**Affiliations:** 1 Santa Casa de Misericordia de São Paulo, São Paulo, Brazil

## Background

It is a common practice to administer basal insulin after oral diabetes agents fail as a first step in insulinization. However, we do not know which regime is better regarding glycemic control: adding basal insulin to sulfonylurea or stopping sulfonylurea and starting a basal bolus regime.

## Objective

To compare glycemic variability in T2D patients being treated with two different regimes of diabetes treatment: basal insulin plus sulfonylurea versus basal bolus.

## Materials and methods

A retrospective study of a cohort of 51 T2D patients. To evaluate glycemic variability, we collected data from glucometers (accu check 360 software was used to download the data) of all patients that came for physician's appointments at a public hospital between March and June 2015. We analyzed data of mean capillary glucose and its variability (standard deviation) from the 90-day period preceding the final download date. Glycemia and hba1c were also used for this analysis. Chi-square tests and student's t-test were performed for statistical analysis, where p < 0.05 was considered significant.

## Results

**Figure 1:**
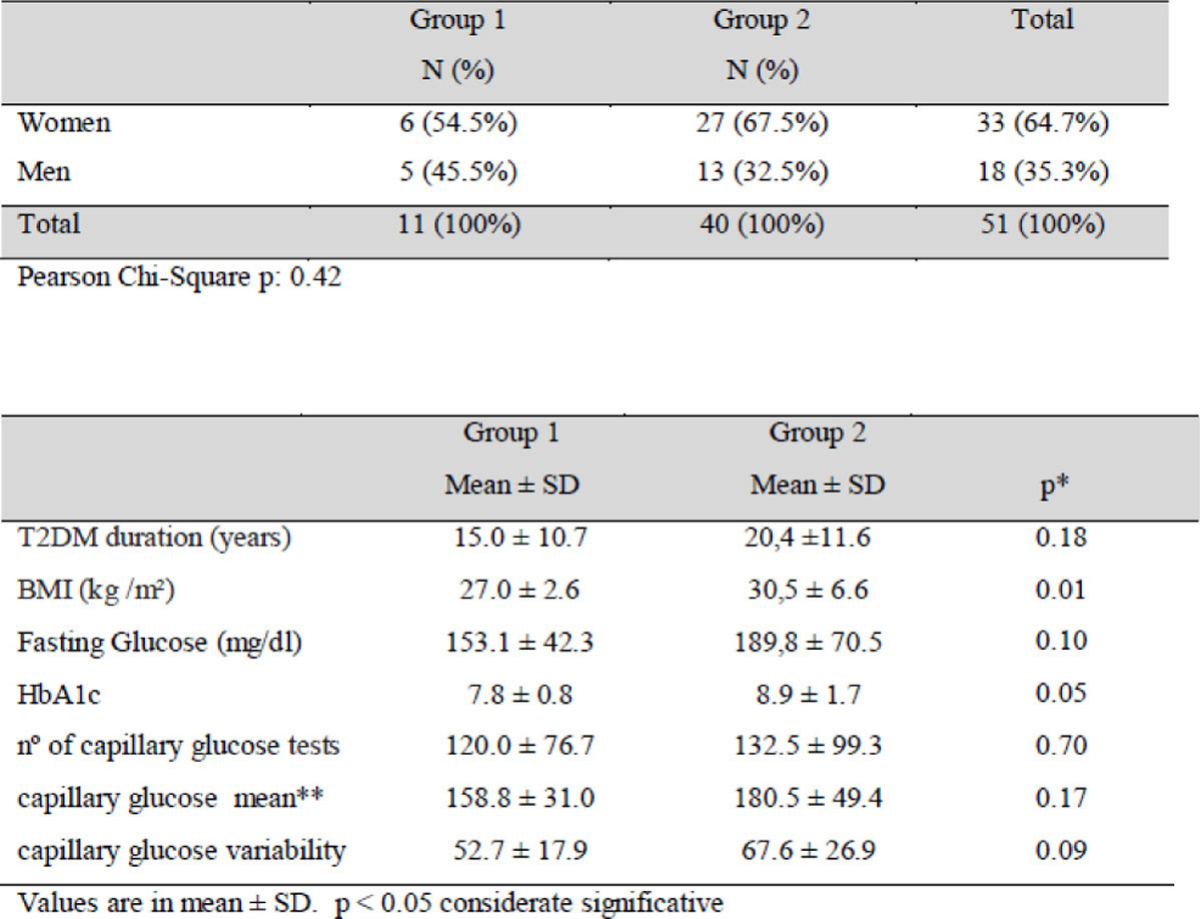
Results for both groups.

Of the 417 patients included in this study, 51 were eligible for analysis. 11 patients were using insulin plus sulfonylurea (group 1) and 40 were using basal bolus treatment (group 2). The proportion of men and women in each group was 45.5%: 54.5% in group 1 and 32.5%: 67.5% in group 2 (Pearson chi-square p=0.42). There was no difference between treatments regarding A1c and glucose variability (Figure 1).

## Conclusion

Sulfonylurea plus basal insulin and basal bolus had the same glucose control and glycemic variability in both patient sample groups.

